# Efficacy and Safety of Oral Valiltramiprosate in APOE4/4 Homozygotes with Early AD: Topline Results from the APOLLOE4 Phase 3 Trial

**DOI:** 10.1002/alz70861_108821

**Published:** 2025-12-23

**Authors:** Aidan Power, Susan Abushakra, David Watson, Anton P. Porsteinsson, Marwan N. Sabbagh, Emer MacSweeney, Mercè Boada, Earvin Liang, Susan Flint, Rosalind MacLaine, Patrick Kesslak, John A. Hey, Adem Albayrak, Arthur Rosenthal, Martin Tolar

**Affiliations:** ^1^ Alzheon, Inc., Framingham, MA USA; ^2^ Alzheimer's Research and Treatment Center, Wellington, FL USA; ^3^ University of Rochester School of Medicine and Dentistry, Rochester, NY USA; ^4^ Barrow Neurological Institute, Phoenix, AZ USA; ^5^ Re‐Cognition Health, London, Greater London UK; ^6^ Research Center and Memory Clinic, ACE Alzheimer Center Barcelona, Universitat Internacional de Catalunya, Barcelona Spain; ^7^ Alzheon Inc., Framingham, MA USA; ^8^ Alzheon, Framingham, MA USA

## Abstract

**Background:**

Valiltramiprosate (ALZ‐801) is an oral inhibitor of amyloid oligomer formation. Tramiprosate (active agent in ALZ‐801) had shown promising efficacy signals with favorable safety in ∼1300 APOE4 carriers with AD, with no observed ARIA‐E. This trial evaluated Valiltramiprosate in APOE4/4 homozygotes with Early AD.

**Method:**

This 78‐week double‐blind, placebo‐controlled, two‐arm trial that randomized 325 homozygotes (162 to placebo, 163 to 265 mg BID), stratified by MCI (MMSE 27‐30) or Mild AD (MMSE 22‐26). MRI (1.5/3 Tesla) were conducted every 26 weeks and analyzed by Clario Inc. The clinical outcomes were ADAS‐Cog13 (primary), CDR‐SB (key secondary) and DAD (disability assessment for dementia, secondary). Hippocampal volume (HV) was the main imaging outcome. MMRM was the primary analysis model.

**Result:**

Safety population (*N* =325) was 51% females, 90% Caucasian, mean age 68 years, MMSE 25.6, 39% with MCI (MMSE 27‐30) and 30% with baseline microhemorrhages and/or siderosis. In the full analysis set (*N* =320), the placebo‐adjusted least‐square mean (LSM) difference in change from baseline (CBL) on ADAS‐Cog13 favored drug non‐significantly (delta=‐0.50; *p* =0.61, 11% vs placebo CBL); CDR‐SB and DAD numerically favored drug by 23% and 29%, but HV favored drug significantly (74 mm^3^, nominal *p* =0.017, *18% less atrophy*). Prespecified Mild AD showed small nonsignificant clinical effects favoring placebo, but showed numerical benefit on HV (51mm^3^, 12% *p* = 0.115). In prespecified MCI, all outcomes favored drug (nominal *p* ‐values): ADAS‐Cog13= ‐2.14 (*p* =0.041; 52%); CDR‐SB= ‐0.65 (*p* =0.053, 104%); DAD= 6.09 (*p* =0.016, 96%); and HV= 108 mm^3^ (*p* = 0.004, 26% less atrophy). Nausea (mostly mild) was the most common adverse event; incidence of ARIA‐E was the same as placebo.

**Conclusion:**

In the overall population, ADAS‐Cog13 did not achieve significance, but the hippocampus showed significant 18% slowing of atrophy. The pre‐specified Mild group showed trends to HV atrophy slowing that did not translate to clinical benefits. The pre‐specified MCI group showed significant 28% HV atrophy slowing with meaningful cognitive and functional benefits and positive trends on several secondary clinical outcomes. Overall safety was favorable with no increased ARIA. In the high‐risk APOE4/4 population, this positive benefit‐risk profile supports valiltramiprosate’s potential as an oral disease‐modifying treatment for APOE4/4 homozygotes with MCI.

